# Solidification/Stabilization of Chromium-Contaminated Soils by Polyurethane during Freeze–Thaw Cycles: Mechanical, Leaching and Microstructure Characterization

**DOI:** 10.3390/ma17061347

**Published:** 2024-03-15

**Authors:** Qiang Ma, Pangkun Zheng, Junjie Chen, Xuesong Lu

**Affiliations:** 1 School of Architectural Engineering, Huanggang Normal University, Huanggang 438000, China; 20121012@hbut.edu.cn; 2 Hubei Provincial Ecological Road Engineering Technology Research Center, Hubei University of Technology, Wuhan 430068, China; 102110933@hbut.edu.cn (P.Z.); chenghongliang@hbut.edu.cn (J.C.); 3 Huanggang Ecological Architecture and Renewable Resources Research Center, Huanggang 438000, China

**Keywords:** freeze–thaw cycle, hexavalent chromium-contaminated soil, solidification/stabilization, toxicity leaching ability, soil porosity, FTIR spectroscopy

## Abstract

The treatment of chromium-contaminated soil in seasonal frozen soil areas has been the subject of recent interest. Polyurethane (PU), as a polymer material with excellent freeze–thaw resistance and abrasion resistance, has the potential to solidify Chromium-Contaminated soil in seasonal frozen soil areas. However, there is a lack of research on the mechanism of PU involved in solidifying/stabilizing chromium-contaminated soil in seasonal frozen regions from the perspective of pore structure and functional group coordination bonds. In this study, the leaching behavior of PU with different contents under different freeze–thaw cycles was analyzed, and the mechanism of PU in seasonal frozen regions was explored from the perspective of pores and functional groups by combining various microscopic characterization methods. The results show that PU can effectively resist the deterioration of chromium-contaminated soil after freeze–thaw cycles and can better prevent the harm of secondary leaching. The leaching concentration of chromium ion is only 1.09 mg/L, which is below China’s regulatory limits. PU is beneficial for inhibiting the expansion of ice crystals in chromium-contaminated soil in seasonal frozen soil areas. PU solidifies chromium by physical encapsulation and complexation reactions. The amide functional groups, methyl-CH_3_ and isocyanate groups in PU play a leading role in the complexation with chromium. Although the freeze–thaw cycle will destroy the coordination bond between the PU functional group and chromium, chromium cannot break through the bond of PU film. This study confirmed the feasibility of using PU to solidify Chromium-Contaminated soil in seasonal frozen soil areas, which can provide research support and reference for in situ engineering in the future.

## 1. Introduction

Chromium ion pollution is more serious in seasonal frozen soil areas, and Cr(VI) is more harmful [1,2]. The chromium ions are from natural [3] and artificial environments [4] (e.g., industrial production waste, leather products). The freeze–thaw cycle occurs mainly in seasonal regions; the formation of ice causes volume expansion. In a finite volume, ice crystals expand during formation [5]. During freezing, the development of ice crystals squeezes the soil particles, expanding the pores between the soil particles. During thawing, the expanded pores cannot be recovered. Hence, the process of the freeze–thaw cycle will alter the structure and arrangement of soil particles, changing the mechanical and physical characteristics of the soil [6]. This may aggravate the risk of chromium migration and leaching in seasonal frozen regions. On the other hand, the adsorption and desorption of heavy metal ions will be affected during the freeze–thaw cycle. Based on this, controlling hexavalent chromium-contaminated soil in seasonal frozen areas may be more difficult. So, how to solidify chromium ions in seasonal freezing soil to prevent their diffusion is of great research value.

At present, taking into consideration the engineering cost and remediation efficiency, solidification/stabilization is an economic and widely applicable remediation measure. Pollutants are prevented from migrating and diffusing into the environment, reducing their toxicity and transfer rate [7]. Although the excellent solidification capability and mechanical properties of Portland cement have been proven [8], the use of these materials cannot meet the requirements of long-term stability in seasonal frozen regions. It is not environmentally friendly to use various materials, such as cement. Chen et al. [9] used nanoscale zero-valent iron, which is easy to prepare but susceptible to temperature, as a reducing agent. Therefore, the redox method is unsuitable for in situ chromium treatment in frozen soil areas. Most of these methods are expensive and have the disadvantages of incomplete removal of heavy metals and high reagent consumption. Therefore, scholars began to explore the application of low-cost solid waste materials in heavy metal-contaminated soil areas. Mitali Nag et al. [10] used pozzolanic bottom ash to solidify heavy metals at room temperature, but its dependence on an alkaline environment limits its application in seasonal frozen soil areas. Zhang et al. [11] used alkali-activated zeolite/MSWI fly ash to explore the effect of different temperatures on the solidification of heavy metals. Komaei, A. et al. [8] explored the potential of alkaline-activated slag to stabilize and solidify arsenic-contaminated soil. The requirements of an alkaline environment for materials such as ash and inexpensive pozzolanic agents [12] will limit their application in seasonal frozen soil areas, and the durability of solidified heavy metals in freeze–thaw areas is a concern. In order to promote the formation of hydration products and improve their ability to treat contaminated soil, an alkaline environment is essential. The formation of hydration products is affected by temperature, with adverse effects on applications in low-temperature environments. These solid waste-based cementitious materials have high requirements for an alkaline environment, which may increase the risk of the secondary leaching of heavy metal ions. Samadi, P. et al. [13] explored the mechanical strength of geopolymer/cement materials under different curing conditions (curing time and curing parameters) and different binder types, which limits their application in permafrost regions. The use of appropriate materials is particularly important for the treatment of contaminated sites in seasonal frozen soil areas.

Polyurethane (PU) as an organic binder has the advantages of adhesion, fast bonding speed and environmental protection and is extensively employed in engineering [14]. Previous studies have shown that the addition of polyurethane to cement mortar can improve mechanical strength [15]. Li et al. [16] found that the mechanical properties and wear resistance of mortar containing polyurethane under freeze–thaw cycles and sulfate attack are good. This may be able to inhibit the secondary release behavior of heavy metal ions as a result of the increase in soil fissures caused by ice crystal expansion. Moreover, PU is not sensitive to temperature, which may make up for the defects of other materials in seasonal frozen soil areas. Wei et al. [17] found that with the increase of freeze–thaw cycles, the roughness of the polyurethane surface decreased steadily, but its elastic modulus increased. At the same time, some studies have shown that PU can adsorb heavy metal ions in water and convert heavy metals into more stable forms through physical or chemical reactions [18]. Hence, PU has the potential to serve as a novel binder for the solidification/stabilization of soil contaminated with heavy metals. This may be beneficial to address the strength degradation of contaminated soil and the risk of secondary leaching of heavy metals in freeze–thaw areas. However, there are few studies on the leaching and microscopic pore characteristics of PU-solidified chromium-contaminated soil in seasonal frozen soil areas, especially since the mechanism of the combination of PU functional groups and chromium ions is not clear.

The purpose of this study is to explore the curing stability, leaching behavior and microscopic mechanisms of PU under freeze–thaw cycles and develop effective methods to adapt to the solidification of chromium-contaminated soil in freeze–thaw areas to prevent its leaching. In order to verify the feasibility of PU curing chromium-contaminated soil in frozen soil areas, a series of experimental studies were carried out. The toxicity characteristic leaching procedure (TCLP) test and unconfined compressive strength (UCS) test were used to determine the bearing capacity and environmental safety of chromium-contaminated soil after PU remediation under seasonal frozen soil conditions. The binding state of functional groups in PU during the solidification of chromium was determined by Fourier transform infrared spectroscopy (FTIR). Scanning electron microscopy (SEM) and nuclear magnetic resonance (NMR) were used to study the microscopic mechanism of PU curing chromium-contaminated soil under freeze–thaw cycles, including the effect of the PU skeleton on soil pores and the effect on chromium solidification stability. This study verified the feasibility of PU solidifying chromium in seasonal frozen regions and revealed the mechanism by which PU surface functional groups solidify chromium ions during freeze–thaw cycles. The application of PU can promote the reuse of chromium-contaminated land resources and enhance the resistance of leachate erosion in chromium-contaminated sites in frozen soil areas, thereby providing a reference for future in situ engineering.

## 2. Materials

### 2.1. Soil Material

The silty clay used in this study came from Wuhan, China. The soil was ground, screened for soil particle sizes less than 2 mm, and then dried in a 105 °C oven for 24 h to for complete dryness. The initial water content, optimal moisture concentration and maximum dry density of soil were identified according to standards ASTM D4959-16 [19] and ASTM D698-12 (2021) [20]. The pH of the soil was tested in accordance with ASTM D4972-01 [21], as shown in Table 1 [22].

To examine the mineral makeup of the test clay, X-ray fluorescence spectroscopy (XRF) was utilized. The XRF instrument was a Japan Rigaku ZSX Primus III+ (was sourced from Showima City, Tokyo, Japan), and the operating voltage and current were 50 kV and 60 mA, respectively. The soil was ground and passed through a 0.075 mm sieve, dried, and subjected to XRF testing. Uncontaminated clay does not contain chromium; the mineral composition of the soil specimen is listed in Table 2 [22].

PU has the effect of cementation, compaction and encapsulation, which will cause the structure of solidified clay to be denser. To achieve enhanced curing stability, it is advisable to raise the moisture concentration to align with the optimal moisture concentration for the test clay. During the manual preparation of chromium-contaminated soil, it was observed that the optimal moisture concentration slightly exceeded the test requirements. Consequently, preliminary testing indicated that the targeted moisture concentration should be set at 10%.

### 2.2. Polyurethane Materials

Polyurethane (PU) is a commonly used organic gel material in the construction field, known for its bonding properties, environmental protection and rapid bonding ability [14]. According to the type of soft segment used, it can be divided into polyether PU and polyester PU. Polyether PU has excellent corrosion resistance [15], excellent moisture permeability and strong adhesion. When exposed to complex pollution conditions, polyether PU can maintain stability over a long period of time. Hence, polyether PU was used in this study. Before use, PU was obtained from the full mixing of polyether polyol (liquid A) and polyisocyanate (liquid B). The detailed material parameters and preparation process of polyurethane are similar to those of Ma et al. [22].

### 2.3. Specimen Preparation

Currently, researchers primarily utilize the appropriate salt solution to prepare contaminated clay specimens. Therefore, potassium dichromate [23] was used to study chromium-contaminated soil in this paper.

The method of artificially preparing contaminated clay was adopted in this study: A cylindrical mold with a diameter of 38.1 × 76 mm was used in this study. The clay specimen’s weight was determined based on the mold size and the clay specimen’s density. The amount of water needed was computed in accordance with the moisture concentration. The amount of water needed was determined based on the set water content. Quantitative potassium dichromate was added to the prepared distilled water. Clay specimens and the potassium dichromate solutions were mixed uniformly. To ensure that the moisture in the clay specimens was more homogeneous, the blended soil specimens were wrapped with cling film and placed at 20 °C for 24 h. The foaming of PU is fast, and these foams will have a certain strength within 10 min. Therefore, a specific quantity of PU was added to the clay and thoroughly mixed for a brief duration to ensure high fluidity. The stirring time is normally within 60 s for each specimen. Finally, the artificially chromium-contaminated soil and PU were pressed into the mold with a jack, and it was jacked out after a minute of static. After preparation, the specimens were stored at 20 °C and 95% humidity for the designated curing period.

### 2.4. Test Program

#### 2.4.1. Freeze–Thaw Cycle

The freezing and thawing control panel of Shanghai Husheng, Ltd. (Shanghai, China), was used to perform the freeze–thaw cycle experiments. The temperature range of the test instrument was −30 °C~30 °C. The temperature range of this study was set at −20 °C~20 °C. The freezing rate and thawing rate during the test were set to 1 °C/min. The specimen was maintained at a size of 38.1 × 76 mm with a two-layer plastic film-wrapped specimen. After curing for 7 d, the clay specimen was put into the instrument. In this study, every freezing and thawing process consists of a 12 h freezing period (−20 °C) and a 12 h thawing period (20 °C). Based on the annual weather in China’s seasonal frozen regions, the freeze–thaw cycle’s temperature was chosen. A total of 14 freeze–thaw cycles were performed, and specimens of 0, 2, 4, 6, 8 and 14 cycles were selected to investigate the relevant properties.

#### 2.4.2. UCS Test

The unconfined compressive strength (UCS) test was used to investigate the mechanical properties of the test specimens solidified by PU when experiencing the process of freezing and thawing. This represents one of the most straightforward and efficient tests for assessing the strength of solidified specimens [24]. The effect of varying freezing and thawing processes and PU dosage on the UCS of the test specimens was studied. The UCS test based on ASTM D2166 [25], electronic universal testing equipment with microcomputer control, was used for testing. The maximum capacity of the UCS test system was 20 kN, and the applied strain rate was 1 mm/min. In accordance with the national standard for heavy metal pollution states, the concentration of chromium was 2000 mg/kg [1] to represent the concentration of chromium-contaminated soil in China. According to the pre-experiment, the most suitable curing time is 7 days. After curing, the clay specimen was put into the testing machine. UCS measurements of the specimens were taken after 0, 2, 4, 6, 8 and 14 freeze–thaw cycles.

#### 2.4.3. TCLP Test

TCLP experiments followed the standard ASTM Method 1311-TCLP [26], ASTM C1308-21 [27]. After drying at 50 °C, 5 g of the clay samples in the unconfined experiment was ground and screened for soil particle size less than 0.95 mm, and the ground clay was mixed with 96.5 g of distilled water. By employing a pH tester (HC3800sc, was from Shanghai, China), the solution’s pH was identified to be 3.8 (<5). Hence, the extraction solution was decided upon in accordance with the standard as a combination of glacial acetic acid (also known as CH_3_COOH) and hydroxide of sodium. A total of 64.3 mL of 1 mol/L solution of sodium chloride and 5.7 mL of glacial acetic acid solution were combined. Distilled water was added to the mixture to make 1000 mL. A 1 mol/L sodium hydroxide solution was used to achieve a pH value of the mixture of 4.93, with 0.05 precision. We mixed the prepared 1000 mL extraction solution with 50 g ground clay and placed it into the oscillator. After 18 h of flipping oscillation at a rate of 30 r/min, a 0.45 μm microporous filter was applied to extract 15 mL of the supernatants. A flame absorption atomic analyzer SHIMADZU AA-6880 (was from Shanghai, China) was used to measure the chromium contamination. Sample characteristics are presented in Table 3. P refers to PU dosages (%), and F represents the number of freeze–thaw cycles.

#### 2.4.4. SEM Test

The nature of the changes in the structure of soil solidified/stabilized under freeze–thaw cycle conditions can be evaluated by observing the micromorphology of soil particles. Similarly, the curing effect of polyurethane on chromium can also be judged by observing the binding form between polyurethane and soil particles. A high-resolution field emission scanning electron microscope from Hitachi (SU8010, was from Tokyo, Japan) was used to investigate the sample microstructure. The acceleration voltage of the instrument was 0.1 kV~30 kV, and the maximum beam current was 200 nA. In the unconfined experiment, the samples were ground, and soil particles with flat upper surfaces were selected as test specimens. Prior to usage, these samples were placed in a freeze-dryer, subjected to 48 h of vacuum drying, and sputter-coated with gold.

#### 2.4.5. NMR Test

Soil pore distribution is a pivotal factor affecting soil strength. To explore how freeze–thaw cycles impact soil pore distribution and clarify the mechanisms behind variations in soil strength, a nuclear magnetic resonance analyzer (MicroM12-025, was from Wuhan, China) was employed to examine alterations in soil pore distribution. This NMR instrument has a resonance frequency of 12 MHz and a magnetic field strength of 0.52 T. The previously prepared contaminated soil was introduced into a cylindrical mold 18 mm in diameter and 30 mm in height. Following a 7-day curing period, the sample underwent saturation under back pressure and subsequent drying. The specimen was then put into the nuclear magnetic resonance analyzer for testing.

#### 2.4.6. FTIR Test

To explore the capacity of PU functional groups solidifying chromium in soil, a Fourier transform infrared (FTIR, Nicolet iS 50, was from Wuhan, China) spectrometer was used [28]. The spectral resolution of the FTIR spectrometer was 0.09 cm^−1^, and the spectral range was 50–12,000 cm^−1^. After undergoing freeze–thaw cycles, specimens were ground. Using a 2 mm sieve, the ground samples were processed. After screening, the samples were sent for testing.

## 3. Results and Discussion

### 3.1. UCS Analysis

**Figure 1:**
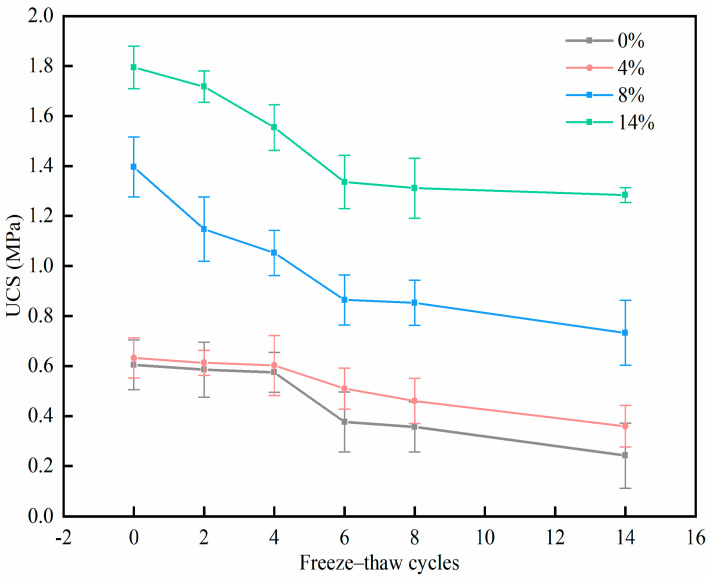
Strength damage of specimens subjected to different freeze–thaw cycles.

As depicted in Figure 1, after 14 freeze–thaw cycles, the strength of all specimens decreased significantly. After the same freeze–thaw cycles, the higher the PU content, the higher the specimen strength. In addition, this study found that the UCS of the specimen containing 4% PU did not increase significantly in the freeze–thaw cycle test compared with the specimen without PU. The UCS of the specimens containing 8% PU and 12% PU increased significantly after the freeze–thaw cycles. In particular, UCS decreased rapidly with a rise in the count of freeze–thaw cycles before 6 cycles in the specimens containing 8% PU or 12% PU. After 6 freeze–thaw cycles, the decline of UCS showed a slowing trend. The UCS of the specimen without PU was only 0.247 MPa after 14 freeze–thaw cycles. The specimen with 0% PU, as shown in Figure 1, was obviously affected by the freeze–thaw cycles, and the strength deteriorated. The specimens containing PU experienced deterioration as a result of the freeze–thaw cycles. But, the strength continued to meet the needs of practical engineering. When the dosage of PU was 12%, the UCS reached 1.284 MPa after 14 freeze–thaw cycles, which is 5.2 times higher than that without PU. The increase in strength may be due to the following reasons: Firstly, during the solidification, the clay particles are bonded by PU due to its strong bonding effect. This bonding effect generates a quasi-cohesion, further enhancing the strength of the specimen. As the PU content increases, the quasi-cohesion between clay particles and PU is stronger [29]. Secondly, the solidification process is accompanied by a PU volume expansion. The inner pores of the soil particles are filled with PU, and the porosity of the clay is reduced. The influence of PU on the pore structure characteristics of soil in frozen soil areas is analyzed in Section 3.4 of this manuscript. Hence, the UCS of the clay specimens increases continuously with the increase in PU content.

The strength damage efficiency of the different freeze–thaw cycles is shown in Equations (1) and (2):

(1) $$R_{t}=\frac{S_{14}{-S}_{0}}{S_{14}}\times 100\%$$

(2) $$R_{a\text{-}b}=\frac{S_{a}{-S}_{b}}{S_{14}{-S}_{0}}\times 100\%$$

where *R_a-b_* (%) refers to the strength damage efficiency of the different freeze–thaw cycles. *S_a_* refers to the UCS with different freeze–thaw cycles. Subscripts *a* and *b* denote different cycles, and subscript *t* refers to total strength damage efficiency.

**Figure 2:**
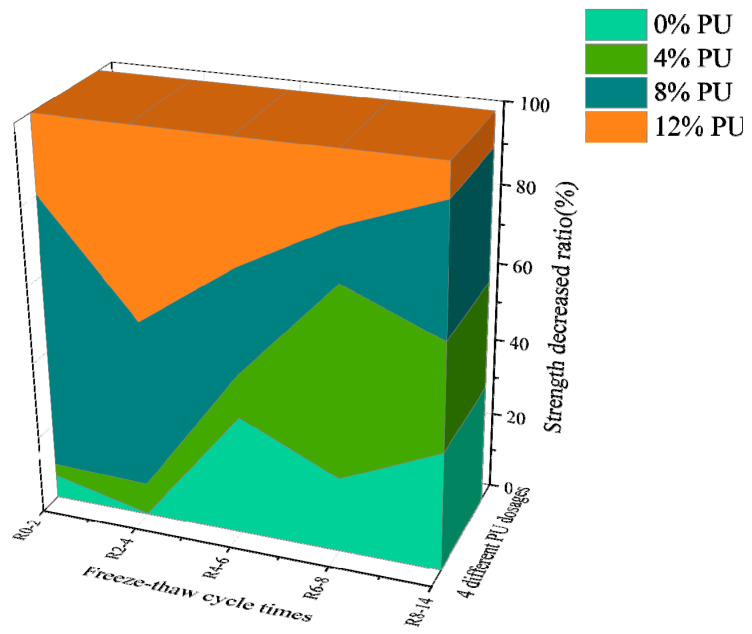
Strength damage efficiency of specimens subjected to different freeze–thaw cycles.

Figure 2 shows the strength loss rate of different freeze–thaw cycles and different PU contents. The four different colors in the figure represent four different contents of PU, and the length of each color on the longitudinal axis indicates the strength loss rate. As Figure 2 illustrates, the strength loss due to the first eight freeze–thaw cycles is the largest. After that, the strength degradation caused by freeze–thaw cycles gradually weakened. After 8 freeze–thaw cycles, the solidified soil specimens showed better frost resistance with an increase in PU content. For example, the *R*_8-14_ of the specimen without PU was 30%, and *R*_8-14_ was only 6% when the PU dosage was 12%. The *R_t_* of the four different PU dosages is 59.70%, 56.20%, 54.10% and 28.26%, indicating that PU may significantly improve soil frost resistance. The main reason for the weakening of specimen strength due to freeze–thaw cycles is that, during freezing, the pore water changes from liquid to solid, and the volume increases. Thus, the soil particles are squeezed, making the pore volume larger and destroying the initial structure of the soil. During melting, the pore water changes from solid to liquid, but the pores in the soil cannot be restored. As the number of freeze–thaw cycles grows, the internal microstructure of the soil continues to be destroyed, and the strength of the soil gradually decreases. After a high number of freeze–thaw cycles, because the internal pore structure is difficult to restore to this original state, the PU-wrapped soil pore structure gradually forms a new stable structure, illustrating a progression from destruction to reconstruction [30]. As a result, the strength degradation gradually diminishes during the 8-14 freeze–thaw cycles. In the meantime, considering the engineering costs and mechanical properties, 8% PU may be the optimal content. However, the optimal content of PU cannot be determined only based on the UCS test; the effect of PU on the leaching concentration of Cr(VI) is also crucial.

### 3.2. The Leaching Behavior of Hexavalent Chromium

**Figure 3:**
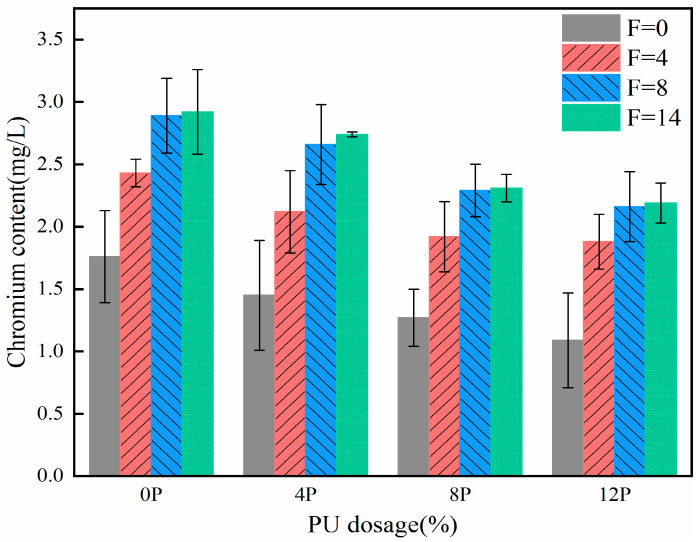
The effect of freeze–thaw cycles on leaching concentration of chromium.

The TCLP results reflect the leaching behavior of chromium-contaminated soil solidified with PU. The specific test steps are given in Section 2.4.3 for the TCLP test. Figure 3 illustrates the effect of freeze–thaw cycles on the leaching concentration of chromium. There is a positive correlation between the number of freeze–thaw cycles and the leaching concentration of chromium under all four PU contents. This suggests that the freeze–thaw cycle negatively impacts the PU-solidified chromium-contaminated soil. As the PU content increases, the leaching concentration of chromium decreases gradually; the leaching concentration of the 12P-0F specimen is only 1.09 mg/L. The addition of PU significantly reduces the leaching concentration of chromium. This indicates that PU effectively solidifies chromium in soil, and increasing the PU dosage enhances the solidification effect. However, the formation of ice crystals during freeze–thaw cycles can cause volume expansion, potentially disrupting the coordination and covalent bonds formed between PU and chromium. It may lead to secondary leaching of chromium. Analysis is provided in the NMR and FTIR sections. According to the thermodynamic principles of soil adsorption, temperature changes can affect the specific and non-specific adsorption of chromium by soil particles, leading to secondary leaching [31].

During the freeze–thaw cycle, part of the chromium is transformed from the bound to the free state. The solidified chromium is released into the soil again. The secondary leaching of chromium is calculated by Equations (3) and (4):

(3) $$L_{T}=L_{14F}-L_{0F}$$

(4) $$L_{aF\text{-}bF}=L_{bF}-L_{aF}$$

where subscripts *a* and *b* refer to different cycles, and *n* refers to PU dosage. *L_T_* refers to the total secondary leaching of chromium, and *L_aF-bF_* refers to the secondary leaching concentration of chromium of the different freeze–thaw cycles.

**Figure 4:**
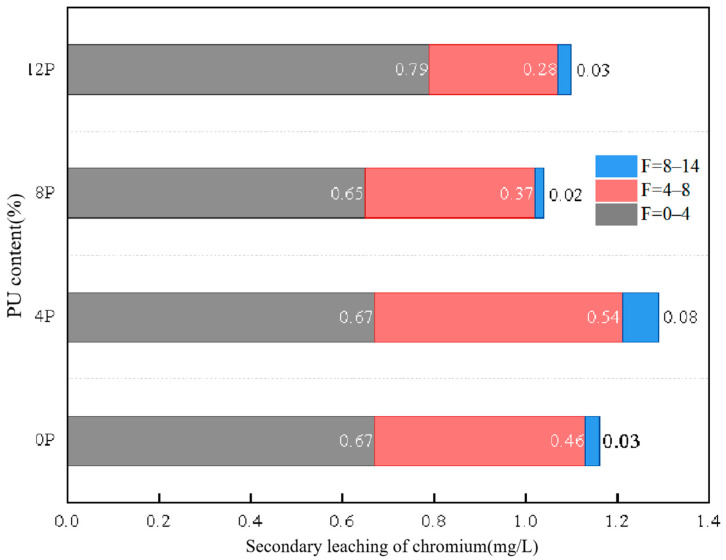
The effect of freeze–thaw cycles on secondary leaching concentration of chromium.

During the early stages of the cycles (F = 0–4), the secondary leaching concentration of chromium grows quickly as the number of freeze–thaw cycles increases (as illustrated in Figure 4). The *L*_0*F*-4*F*_ under four PU dosages is 0.67 mg/L, 0.67 mg/L, 0.65 mg/L and 0.79 mg/L. However, the secondary leaching concentration of chromium decreased with the increase in freeze–thaw cycles (F = 4–8 in Figure 4). This may be because during the freeze–thaw cycle, the wrapped structure formed by PU in the soil stabilizes, preventing the re-diffusion of wrapped chromium ions into the soil. This indicates that PU inhibits the re-diffusion of chromium ions in the soil during freeze–thaw cycles, demonstrating its beneficial effects on frozen soil areas. This indicates that the rapid solidification characteristics of PU are also applicable to frozen soil areas. Moreover, PU will form a stable structure wrapped with chromium in the soil and reduce the risk of secondary leaching. This is much lower than the secondary leaching concentration of chromium solidified by other inorganic cementitious materials, such as cement. In the micromechanism section, the effect of PU on the encapsulation of heavy metal ions and its effect on soil pores will be analyzed. The concentrations of *L*_8*F*-14*F*_ under four PU dosages are 0.03 mg/L, 0.08 mg/L, 0.02 mg/L and 0.03 mg/L. Therefore, it can be inferred that the leaching concentration of chromium remains relatively constant as the number of freeze–thaw cycles increases. With increasing freeze–thaw cycles, the soil with PU tends to reach a stable condition in terms of its composition, structure and characteristics. Hence, the leaching concentration of chromium tends to stabilize. The secondary leaching concentration of specimens with 8% PU was the lowest, indicating better mechanical properties or secondary leaching results in samples containing 8% PU. Thus, 8% PU was determined to be the optimal dosage.

Based on the unconfined compressive test and TCLP test results, when the specimen underwent identical rounds of freezing and thawing, the specimen strength increased, and the chromium leaching rate decreased with increasing PU content. The concentration limit of leached pollutants for chromium in domestic waste landfills in China is 1.5 mg/L. Hence, PU can effectively reduce the leaching concentration of chromium-contaminated soil and prevent the deterioration of strength and solidification ability after freeze–thaw cycles.

### 3.3. Micromorphology Analysis of PU

**Figure 5:**
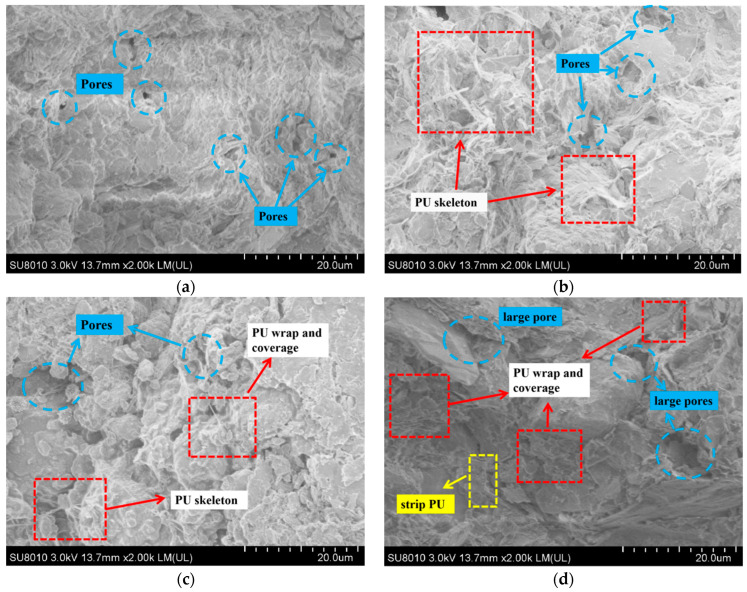
Microstructure of sample: (**a**) 0PU-0F, (**b**) 8PU-4F, (**c**) 8PU-8F and (**d**) 8PU-12F.

The external surface morphology of the specimen was observed by the SEM analysis to interpret the mechanism of damage caused by freeze–thaw cycles and the PU solidification mechanism. According to the above discussion, 8% PU in the specimen is the optimal dosage. Figure 5 illustrates the microstructural features of clay soil after solidification by 8% PU after 0, 4, 8 and 14 freeze–thaw cycles. In (a), (b), (c) and (d) of the SEM results, the blue area represents the pore, and the red area represents the skeleton of the PU and the structure that wraps and covers the contaminated soil particles. The yellow area represents a strip-shaped PU (possibly caused by freeze–thaw cycles). From Figure 5a, it is evident that the soil particles have undergone no freeze–thaw cycles are densely arranged, and the link of the soil skeleton is mainly plane–plane. An increase in freeze–thaw cycles led to a gradual porosity in the dense soil structure and a notable increase in soil particle cracks [16]. Small pores developed gradually into large pores, and the link of the soil skeleton was mainly point–surface. The phenomenon was probably due to the following reason: The volume of pore water expands after freezing. The envelopment of ice crystals squeezes the soil particles in the process of freezing and thawing. The original stable structure of the soil is disrupted. When the ice crystals melt, the pores cannot restore and become larger. On the contrary, soil particles are squeezed by ice crystals to generate an agglomeration effect [32]. Finally, the redistribution of soil particles and the continuous development of small holes into large holes were observed.

Figure 5b illustrates how the PU created a structure resembling a skeleton between the soil particles, binding them together. When the soil particles endure uneven stress from the outside, they can effectively support the soil to resist external forces. In addition to forming skeletons, PU might build a protective covering of PU film by wrapping around the soil particle surface. This PU film can effectively seal soil particles and heavy metal ions. Hence, the soil particles and heavy metal ions are solidified in the PU film. This is also the main reason for the lower secondary leaching concentration of chromium cured by PU in this study. However, as the number of freeze–thaw cycles rises, the polyurethane “skeleton” is gradually destroyed. The planar structure of PU is dispersed into small blocks and points. This small structure may be the structure of PU-wrapped chromium and soil particles. According to Figure 5d, the PU “skeleton” was almost destroyed, and only strip PU could be seen after 14 freeze–thaw cycles. The specimen’s strength could potentially decline as a result of this. The results of UCS and TCLP tests show that even if the strength of the sample decreases, the secondary leaching concentration is still low. It is possible that a higher number of freeze–thaw cycles affects the overall structure of PU, but the chromium ions in the soil still cannot break through the bondage of the PU coating film. Although the covering effect of PU can always be observed, the surface of PU changes from smooth to irregular. Hence, it is speculated a high number of freeze–thaw cycles may weaken the adhesion and coagulation ability of polyurethane.

### 3.4. Analysis of Pore Structure Characteristics

**Figure 6:**
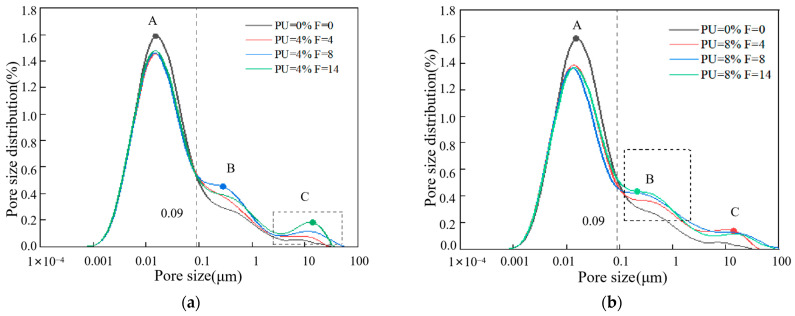
The effect of different freeze–thaw cycles on pore distribution (**a**) when PU content is 4% and (**b**) when PU content is 8%.

Figure 6 demonstrates how freeze–thaw cycles and PU doses impact the distribution of pore dimensions in soil specimens. The signal strength and relaxation time are transformed into pore size distributions (*Y*-axis) and pore size (*X*-axis), respectively, using a systematic inversion tool, where P refers to PU dosages and F refers to freeze–thaw cycles. As before, PU dosages were set to 4% and 8%. The test specimens for freeze–thaw cycles were subjected to 0, 4, 8 and 14 cycles. According to the average size of soil pores in NMR experimental results, pores are classified as small pores (<0.09 μm), mesopores (0.09–1 μm) and macropores (>1 μm). Figure 6a,b shows that the specimens without freeze–thaw cycles only exhibit one peak, which is close the pore size of 0.01 μm. The specimen after freeze–thaw cycles exhibit a second peak, which is near the pore size of 10 μm. The first peak in small pores corresponds to intragranular pores, while the second peak in macropores corresponds to intergranular pores [33]. This may be due to the increase in macropores and intergranular pores between PU-coated chromium-contaminated soil particles caused by freeze–thaw cycles, which also reflects that freeze–thaw may increase the risk of secondary leaching of chromium ions. The results of secondary leaching are shown in Figure 4. Although the freeze–thaw cycle may disperse the structure of PU, the concentration of chromium in the secondary leaching is low because the chromium ions wrapped by PU still cannot break through the PU film. The curve area of the specimen without freeze–thaw cycles is much larger than that of the specimen after the freeze–thaw cycles, when the pore size is less than 0.09 microns. The curve area of small pores (<0.09 μm) decreases after freeze–thaw cycles. This is because small pores or capillary pores are filled with PU-wrapped chromium ions [16]. On the contrary, the curve area of mesopores (0.09–1 μm) and macropores (>1 μm) increases after freeze–thaw cycles. This shows that the number of intragranular pores decreases, while the number of intergranular micropores increases. There is a possible explanation for this result. The development of ice crystals in the pores of the clay soil specimen leads to the extrusion of soil particles, which leads to a gradual decrease in intragranular pores. The PU may wrap around the soil to form aggregates; clay soil particles are also squeezed together to form a clay soil particle aggregate. However, intergranular pores cannot shrink with the melting of ice crystals. Hence, the number of intergranular pores increases, and this may cause a decrease in strength. This can explain the behavior characteristics of the UCS. However, due to the rapid solidification ability of PU, chromium ions are wrapped in the PU film and are not affected by the increase of soil aggregate pores caused by freeze–thaw cycles. This reduces the risk of secondary leaching of chromium ion. This is consistent with the results of the leaching behavior of chromium in this study.

**Figure 7:**
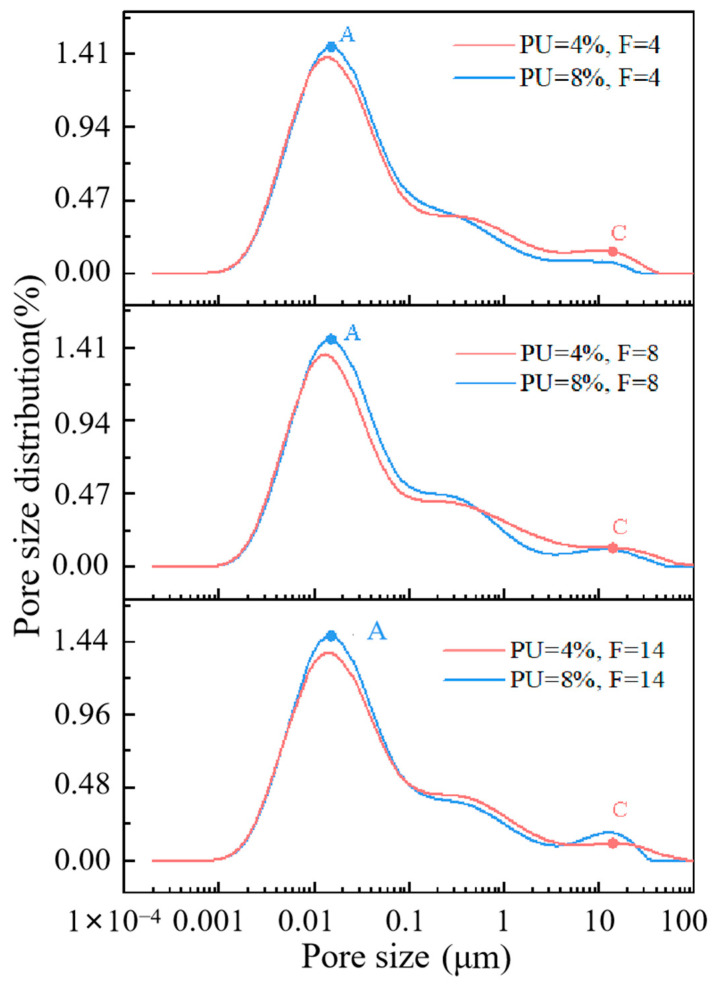
The effect of PU dosage on pore size distribution of soil specimens.

**Figure 8:**
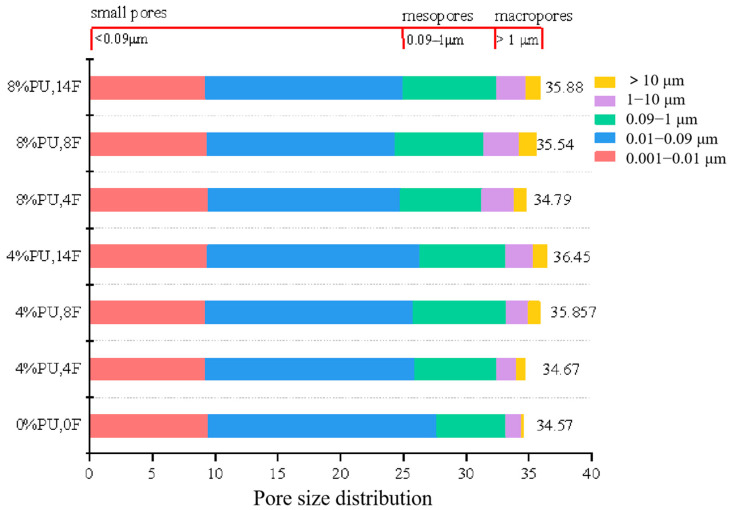
The proportion of pores with different pore sizes.

Figure 7 shows the pore size distributions of soil specimens with different PU dosages. Figure 8 displays the percentage of pores with varying pore diameters. It can be observed that during the same freeze–thaw cycle, PU can decrease the average porosity, reflecting its dense structure. After 14 freeze–thaw cycles, the average porosity is 36.45%, and 35.88% with the polyurethane dosage of 4% and 8%. The higher the polyurethane dosage, the lower the peak value of point A, indicating that polyurethane can significantly reduce the number of small pores (<0.09 μm). After 4, 8 and 14 freeze–thaw cycles, the number of small pores (<0.09 μm) decreased by 1.16%, 1.40% and 1.32%. This result can be attributed to the effect of PU on soil particles, in particular, the effect of PU on the soil small pore structure; chromium ions are present in these small pores. The volume of PU expands to fill small pores in the solidification process of PU. Section 3.3 detailed the ability of PU to wrap and cover the clay soil particles, forming a non-porous polyurethane film on the soil particle’s surface to seal the clay soil particles. This film effectively resists the expansion of ice crystals. These factors contribute to the decrease in mesopores. Moreover, since the PU used in this study is hydrophobic, it can greatly reduce the adverse effects of water from melted ice crystals on the solidification of chromium during the melting process in the soil pores. This property is beneficial for PU in solidifying chromium ions in soil pores in areas with seasonal frozen soil.

**Figure 9:**
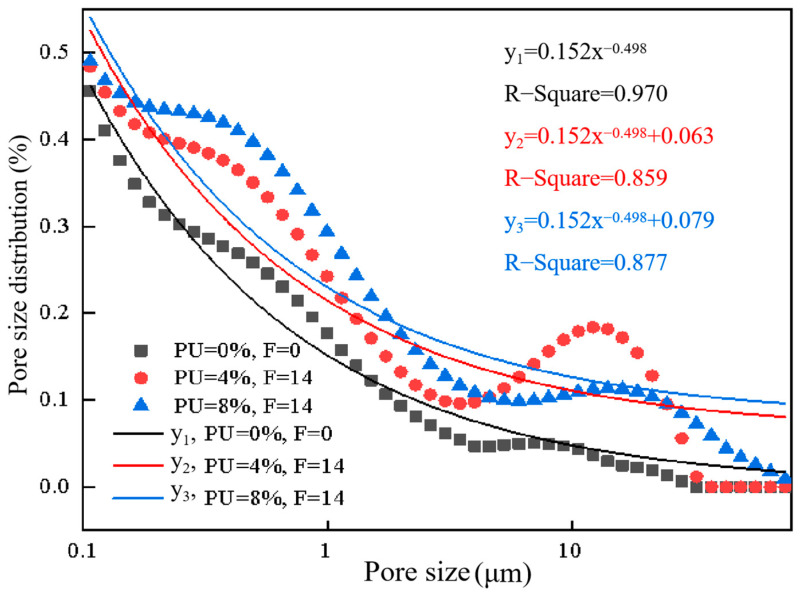
The nonlinear correlation of the specimens 4P-0F, 8P-14F and 8P-14F.

Although the effect of PU on the number of mesopores (0.09–1 μm) and macropores (>1 μm) shows no obvious trend, it can be found that the second peak at point C disappeared when the polyurethane dosage was 8%. The 8P-14F curve is more similar to the specimen with no freeze–thaw cycles. To verify the above results, the mesopores and macropores of 0P-0F, 8P-14F and 8P-14F specimens were chosen as the study subjects. The nonlinear correlation of the specimens was analyzed, as shown in Figure 9. Firstly, the fitting curve of the 0P-0F specimen was set as a benchmark curve for it had not been subject to a freeze–thaw cycle. The curve of 4P-0F fits the following function:

(5) $${y=\mathrm{ax}}^{b}+c$$

where *a* is 0.152, *b* is −0.498, *c* is 0, and the correlation coefficient *R-Square* is 0.970. *a* and *b* in the function are set as constants to simulate the pore diameters distributed in the soil specimens with no freeze–thaw cycles. The correlation coefficient *R-Square* characterizes its nonlinear correlation.

(6) $$R-\mathrm{square}=\frac{\mathrm{SSR}}{\mathrm{SST}}$$

where *SSR* refers to sum of squares of the regression and *SST* refers to the total sum of squares. The value of *R-Square* ranges from 0 to 1. The fitting curve with a large *R-Square* indicates a smaller error of the corresponding points between the fitting data and the original data. As shown in Figure 9, the *R-Square* of *y*_3_ is 0.877, which is better than that of *y*_2_. Hence, the nonlinear correlation of the 8P-14F specimen is better than that of the 4P-14F specimen. The data analyzed here appear to support the assumption that the pore distribution of soil specimens with high PU content is closer to that of soil specimens free of freezing and thawing processes. PU can effectively attenuate the influence of freeze–thaw cycles on pores.

Based on the SEM test results, PU formed a skeleton-like structure between the soil particles, bonding them together effectively to resist external forces. The skeleton structure of PU may be affected in the early stage of the freeze–thaw cycle, leading to an increase in chromium leaching concentration. This could be due to the destruction of the large PU skeleton, as observed in the NMR test, where PU breaks into small pieces and point structures, filling the pores. However, in the middle and late stages of freezing and thawing, it can be seen from the secondary leaching results that the destruction of the PU skeleton did not increase the secondary leaching concentration of chromium. This is because the small block structure of PU formed a stable film structure that solidified chromium and soil particles. Based on the NMR test and the fitted data results, PU could effectively attenuate the negative effects of freeze–thaw cycles on pores. High-PU-content soil specimens have pore distributions that are more similar to those of soil specimens that do not undergo freezing and thawing processes.

### 3.5. Further Solidification Mechanism Analysis

**Figure 10:**
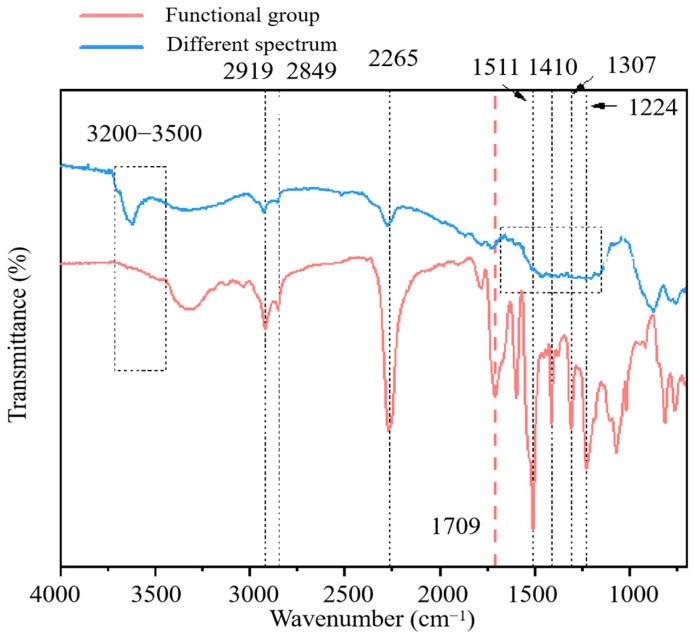
Main functional groups in the complexation with chromium.

The changes in chemical bonds and functional groups [34] in the specimens were analyzed using an FTIR spectrometer (Nicolet iS50, was from Thermo Fisher Scientific, Shanghai, China). The FTIR results of the control group and the PU groups used in this experiment are shown in Figure 10. Clay particles constitute a complex three-phase mixture containing metal oxides such as SiO_2_ and Al_2_O_3_, each with its own distinct infrared spectra. Consequently, this significantly affects the analysis of the characteristic peak spectrum of polyurethane. To reduce the influence of soil particles on the infrared spectrum and simulate the actual engineering conditions, a blank specimen and an 8P-0F specimen were used for differential spectral analysis by the infrared software OMNIC 9.2 (was from Thermo Fisher Scientific, Shanghai, China). The blank specimen was identical to the 8P-0F specimen. The difference spectrum analysis of the two specimens reflects the adsorption activity of polyurethane functional groups on heavy metal chromium in soil. The difference spectrum differs from the spectrum of PU functional group in several important ways. The difference spectrum exhibits several notable differences from the spectrum of the PU functional group. For instance, there is a wide peak at 3200–3400 cm^−1^ in the difference spectrum, corresponding to the free N–H bending vibration and bonded N–H bending vibration. Additionally, peaks at 2919 cm^−1^, 2849 cm^−1^ and 2265 cm^−1^ represent the –CH_3_ asymmetric bending vibration, –CH_3_ symmetric bending vibration and N=C=O asymmetric bending vibration, respectively [35]. There are three significant peaks in the different spectrum, which are more pronounced than the wide peak at 3200–3400 cm^−1^. This suggests that –CH_3_ of the methyl group may play a crucial role in the PU curing process [36]. The –CH_3_ group may preferentially form coordination bonds with chromium ions. Of course, there are inevitably some cations present in the soil environment. Other studies have indicated that ions such as K^+^, Na^+^, Ca^2+^ and Mg^2+^ have no adverse effects on the complexation of functional groups with chromium [37]. The peaks at 1511 cm^−1^ and 1307 cm^−1^ refer to bending vibration N–H of the amide I band and angular vibration N–H of the amide III bands [38]. The peak at 1410 cm^−1^ and 1224 cm^−1^ are assigned to the N–H bending vibration of isocyanate dimer and C–O bending vibration of isocyanate. This shows that the isocyanate group is more active in the complexation process of PU and chromium. There is a wide platform at 1100–1600 cm^−1^ in the difference spectrum, which includes N–H and C–O bending vibration of the ester group [39]. This phenomenon is likely due to the close proximity of these four characteristic peaks, with the shoulders overlapping to form a broad platform. Meanwhile, other peaks show minimal changes, such as the peak at 1709 cm^−1^, which corresponds to the C=O bending vibration. The C=O/C–O groups have been shown to selectively adsorb many heavy metals [40]. In conclusion, amide functional groups, –CH_3_ of methyl, and the isocyanate group play leading roles in the complexity with chromium. The ability of other functional groups to complex chromium is weak. These outcomes are consistent with earlier research [41,42].

**Figure 11:**
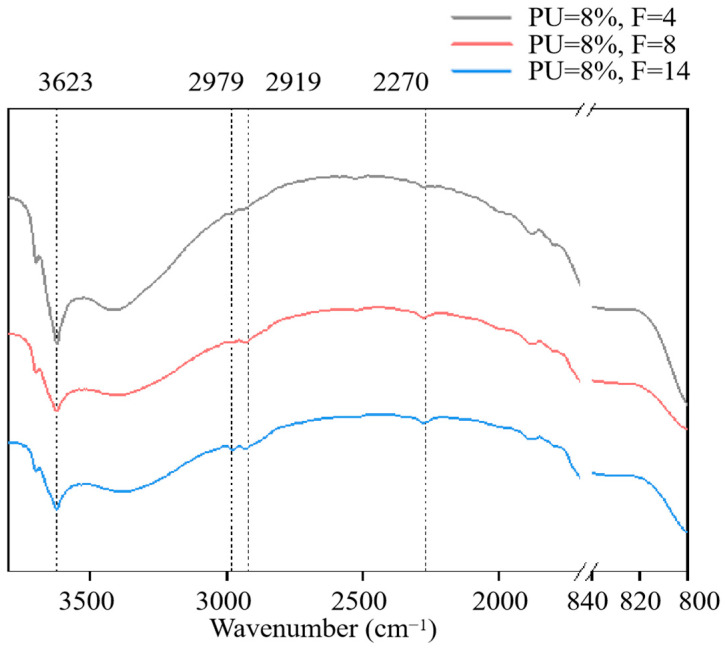
FTIR results of specimens subjected to different freeze–thaw cycles.

As shown in Figure 11, the three curves are the infrared spectra of soil specimens solidified by PU in various freeze–thaw cycles. The spectrum of specimen 8P-4F exhibits the highest intensity at the characteristic peak of 3623 cm^−1^, corresponding to the stretching vibration of –OH in dissociative water molecules. This discrepancy can be attributed to the disruption of the bonds between soil particles and water molecules by the freeze–thaw cycles, leading to the conversion of structural water into free water [43]. The transmittance at 2979 cm^−1^, 2919 cm^−1^ and 2270 cm^−1^ is lowest in the infrared spectrum of specimen 8P-4F. With an increasing number of freeze–thaw cycles, the transmittance at 2919 cm^−1^ (–CH_3_), 2849 cm^−1^ (–CH_3_) and 2271 cm^−1^(N=C=O) rises. These functional groups were demonstrated as highly active coordination sites for hexavalent chromium ions in this chapter. This indicates that freeze–thaw cycles will destroy the coordination bond between chromium and PU functional groups in addition to destroying the soil structure. These results are consistent with Section 3.2 and can explain the leaching behavior characteristics of hexavalent chromium. The application of PU is beneficial in reducing the leaching concentration of chromium under the condition of a freeze–thaw cycle. From the perspective of coordination bonding of functional groups [44], it is found that as the quantity of freeze–thaw cycles rises, the coordination bond formed by polyurethane and hexavalent chromium ions is destroyed, and chromium is re-released into the soil and converted from a bound state to a free state. However, it was found by the SEM test that chromium would not break through the bondage of PU film due to the physical encapsulation of PU (in Figure 5). Therefore, it is important to select the appropriate amount of PU based on the actual temperature conditions of the seasonal frozen soil area where the chromium-contaminated site is located. Each specimen underwent repeated FTIR testing, ruling out the possibility that the observed results are due to chance factors.

The results of SEM and FTIR tests revealed that chromium was solidified by PU through a combination of physical encapsulation and complexation reactions. PU formed a protective layer around the surface of soil particles, effectively sealing them and reducing the diffusion of chromium. Additionally, the amide functional groups, methyl (–CH_3_), and isocyanate groups in PU played pivotal roles in the complexation process with chromium. During the freeze–thaw cycle, due to the adsorption of PU’s physical coating film and functional groups, chromium ions can be solidified and stabilized, avoiding the risk of secondary leaching caused by freeze–thaw cycles. However, actual engineering applications are inherently complex, necessitating the selection of an appropriate polyurethane content based on various factors, such as the temperature range and pollutant concentration of the contaminated site in the seasonal frozen soil area. Therefore, further exploration of PU’s application in solidifying heavy metals in seasonal frozen soil areas should consider factors such as different freeze–thaw cycle temperature ranges, the combined effects of multiple pollutants and variations in pollutant concentrations within seasonal frozen soil areas. 

## 4. Conclusions

This work examined the strength and the leaching toxicity characteristics of PU-solidified chromium-contaminated soil after various freeze–thaw cycles using the UCS and TCLP tests. In addition, the microscopic experiments SEM, FTIR and NMR were used to explain the reasons for the changes in the macroscopic experimental indicators. PU, as a novel solidifying agent, solves the problem of traditional solidifying agents lacking stability in seasonal frozen regions. The following are the primary conclusions:

(1) PU successfully mitigates the strength degradation of chromium-contaminated soil after freeze–thaw cycles. The UCS of specimens with 12% PU reaches 1.284 MPa after 14 freeze–thaw cycles, which is 5.2 times higher than specimens without PU.
(2) PU can effectively reduce the leaching concentration of chromium-contaminated soil and prevent the deterioration of solidification ability after freeze–thaw cycles. The chromium secondary leaching concentration of specimen 12P-14F was only 1.09 mg/L. This is in line with China’s chromium pollution standards.
(3) PU-solidified chromium-contaminated soil in seasonal frozen regions has great potential. The polyurethane film can effectively resist the extrusion of ice crystal volume expansion. In addition, PU can fill soil pores (0.01–0.09 μm), which reduces the secondary leaching risk of heavy metal chromium after freeze–thaw cycles in frozen soil areas. Although freeze–thaw cycles disperse the PU structure, increasing the number of pores sized 1–10 μm, heavy metal chromium remains encapsulated within the PU film, maintaining chromium concentration in toxic leachate within acceptable limits.
(4) PU solidified chromium through physical encapsulation and complexation reaction. PU wrapped on the surface of soil particles, sealing soil particles to reduce the diffusion of chromium. On the other hand, the amide functional groups, –CH_3_ of methyl and the isocyanate-group in PU played significant roles in the complexity with chromium.

The temperature setting of the freeze–thaw cycle in this study has limitations; researchers can consider setting a multi-gradient temperature range. In the actual process application, the amount of PU needs to be considered comprehensively according to the temperature range of the local environment. In practical engineering, the freeze–thaw cycle in seasonal frozen soil area is long term, and the number of freeze–thaw cycles set in this study has limitations. The possibility of PU application in seasonal frozen soil area is preliminarily explored. Further research can focus on the influence mechanism of the functional groups of PU on the conversion of Cr(VI) to less toxic Cr(III). This study did not consider the effect of acid–base environmental conditions on the stabilization of Cr(VI) by PU in seasonal frozen soil areas; the acidic environment may have an adverse effect on the valence state of Cr(VI) converted by functional groups. This needs further research to confirm.

## Figures and Tables

**Table 1:** Main properties of uncontaminated clay specimens used in the experiment.

Properties	Value
Dry density (g/cm^3^)	1.78
Specific gravity (g/cm^3^)	2.69
Optimal moisture concentration (%)	19.5
Initial water content (%)	32.00
Liquid limit (%)	48.20
Plastic limit (%)	20.10
pH	7.43

**Table 2:** Chemical composition of tested clay, wt/%.

Element	SiO_2_	MgO	CaO	Al_2_O_3_	Fe_2_O_3_	K_2_O	Na_2_O	TiO_2_
Soil	63.90	2.19	3.66	18.69	6.45	2.59	0.74	0.95

**Table 3:** Sample properties in tests.

Experiment	Freeze–Thaw Cycle Times						
UCS	PU dosage	chromium concentration 2000 mg/kg					
	0%	0P-0F	0P-2F	0P-4F	0P-6F	0P-8F	0P-14F
	4%	4P-0F	4P-2F	4P-4F	4P-6F	4P-8F	4P-14F
	8%	8P-0F	8P-2F	8P-4F	8P-6F	8P-8F	8P-14F
	12%	12P-0F	12P-2F	12P-4F	12P-6F	12P-8F	12P-14F
TCLP	chromium concentration 2000 mg/kg						
	0%	0P-0F	0P-4F		0P-8F	0P-14F	
	4%	4P-0F	4P-4F		4P-8F	4P-14F	
	8%	8P-0F	8P-4F		8P-8F	8P-14F	
	12%	12P-0F	12P-4F		12P-8F	12P-14F	

## Data Availability

All data are in the paper. The authors confirm that the data supporting the findings of this study are available within the article.
